# Tomato Aqueous Extract Modulates the Inflammatory Profile of Immune Cells and Endothelial Cells

**DOI:** 10.3390/molecules21020168

**Published:** 2016-01-29

**Authors:** Joseph Schwager, Nathalie Richard, Bernd Mussler, Daniel Raederstorff

**Affiliations:** DSM Nutritional Products, P. O. Box 2676, Basel 4002, Switzerland; nathalie.richard@dsm.com (N.R.); bernd.mussler@dsm.com (B.M.); daniel.raederstorff@dsm.com (D.R.)

**Keywords:** chronic inflammation, endothelial dysfunction, human umbilical endothelial vein cells, inflammation, innate immune response, macrophages, nutrients, peripheral blood leukocytes

## Abstract

Nutrients transiently or chronically modulate functional and biochemical characteristics of cells and tissues both *in vivo* and *in vitro*. The influence of tomato aqueous extract (TAE) on the *in vitro* inflammatory response of activated human peripheral blood leukocytes (PBLs) and macrophages was investigated. Its effect on endothelial dysfunction (ED) was analyzed in human umbilical vein endothelial cells (HUVECs). Murine macrophages (RAW264.7 cells), PBLs and HUVECs were incubated with TAE. They were activated with LPS or TNF-α in order to induce inflammatory processes and ED, respectively. Inflammatory mediators and adhesion molecules were measured by immune assay-based multiplex analysis. Gene expression was quantified by RT-PCR. TAE altered the production of interleukins (IL-1β, IL-6, IL-10, IL-12) and chemokines (CCL2/MCP-1, CCL3/MIP-1α, CCL5/RANTES, CXCL8/IL-8, CXCL10/IP-10) in PBLs. TAE reduced ED-associated expression of adhesion molecules (ICAM-1, VCAM-1) in endothelial cell. In macrophages, the production of nitric oxide, PGE_2_, cytokines and ILs (TNF-α, IL-1β, IL-6, IL-12), which reflects chronic inflammatory processes, was reduced. Adenosine was identified as the main bioactive of TAE. Thus, TAE had cell-specific and context-dependent effects. We infer from these *in vitro* data, that during acute inflammation TAE enhances cellular alertness and therefore the sensing of disturbed immune homeostasis in the vascular-endothelial compartment. Conversely, it blunts inflammatory mediators in macrophages during chronic inflammation. A novel concept of immune regulation by this extract is proposed.

## 1. Introduction

Numerous epidemiological studies established the beneficial relationship between a diet rich in fruit and vegetables and health conditions like cardiovascular diseases (CVD), diabetes, obesity, neuro-degeneration and arthritis. Low-grade inflammation is a common feature of these chronic diseases. This is reflected by an unbalanced production of inflammatory mediators including cytokines and chemokines, a disturbed homeostasis of cellular oxidants and mediators of inflammation such as prostaglandins, nitric oxide or the status of the extracellular matrix (ECM). The homeostatic changes influence cell metabolism and alter tissue functions that might favor disease progression [1]. A hallmark of acute inflammation is the increased production of cytokines and chemokines. These enable and enhance inflammatory processes that are essential to recruit immune cells to the sites of inflammation and eliminate pathogens. Some mediators are further required during the resolution of inflammation [2] or for the differentiation of cells that orchestrate the resolution of inflammatory processes such as alternatively activated macrophages [3]. Chronic inflammation maintains a status of un-coordinated production of mediators and metabolites that cause tissue and organ damage. Inflammatory processes have thus an intrinsic dual nature during acute and chronic inflammation. Therefore, substances that modulate the inflammatory status and maintain immune homeostasis in a context-dependent way are expected to prevent the occurrence of health disorders and diseases [4]. In this study we investigated the *in vitro* effects of a tomato aqueous extract (TAE) on inflammatory responses. Previous studies with TAE revealed that it improved the blood flow by reducing platelet adhesion and aggregation [5,6,7]. Likewise, tomato extracts and their lipophilic constituents influenced various mediators of the inflammatory response [8] (reviewed in [9]). In order to cover a potentially wide range of actions in different systemic contexts, we analyzed the effects of TAE in various cellular systems, *i.e.*, human peripheral blood leukocytes (PBLs), human umbilical vein endothelial cells (HUVECs) and macrophage cells (RAW264.7 cells). We found that TAE markedly altered the production of metabolites related to the acute inflammatory response. TAE modulated the transcription factors of the NF-κB signaling pathway and thus regulated expression of inflammatory genes and the production of inflammatory mediators. We show that TAE orchestrates the response of cells to inflammatory stimuli or altered homeostasis in a cell- and compartment-specific way.

## 2. Results

### 2.1. Tomato Aqueous Extract Changed the Inflammatory Profile of Murine RAW264.7 Cells

**Figure 1:**
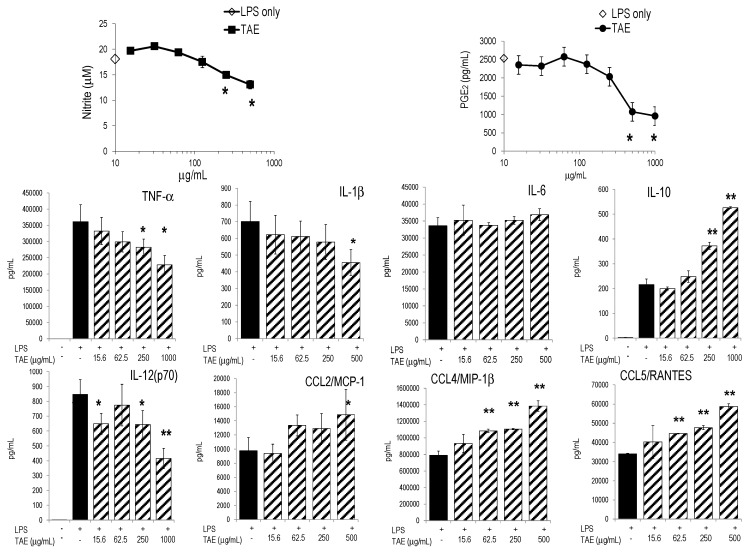
TAE inhibits the production of NO and PGE_2_ and modulates cytokine and chemokine secretion in LPS-activated RAW264.7 cells. Cells were incubated with tomato aqueous extract (TAE), stimulated with 1 μg/mL LPS and cultured for 24 h. “LPS only”: indicates the value obtained from LPS-stimulated cells (without substance) and is indicated on the y-axis. Representative results from one of three independent series are shown as mean (±SD) of duplicate. * *p* < 0.05, ** *p* > 0.01 (*vs.* LPS-stimulated cells). Unstimulated cells produced 0.01 ± 0.00 μM NO and 133 ± 18 pg/mL PGE_2_.

**Figure 2:**
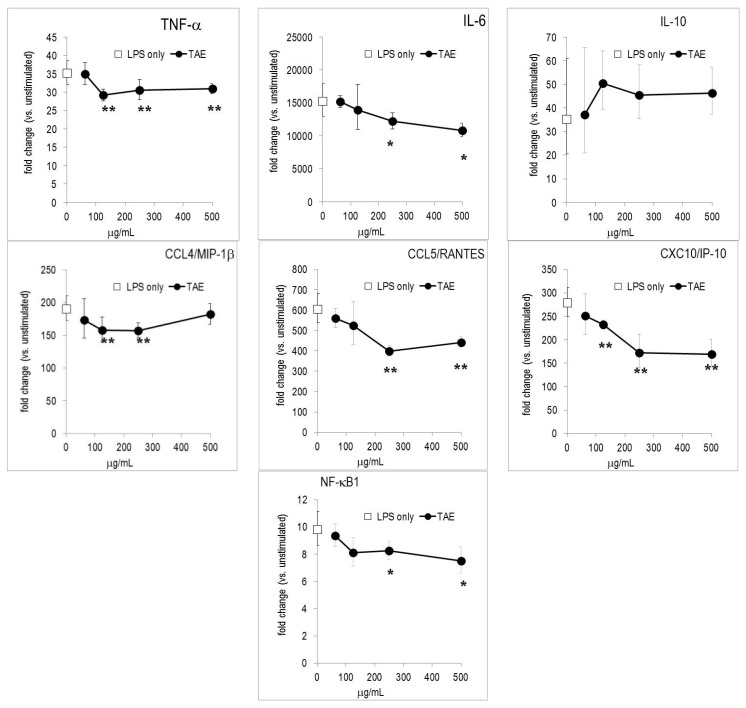
TAE modifies gene expression in LPS-stimulated RAW264.7 cells. Cells were incubated with TAE, stimulated with 1 μg/mL LPS and cultured for 4 h. Gene expression was quantified by RT-PCR and the data expressed as fold change compared to levels observed in unstimulated cells. Mean ± standard errors of duplicates are given. “LPS only”: indicates the value obtained from LPS-stimulated cells (without substance) and is indicated on the y-axis. * *p* < 0.05, ** *p* < 0.01 (*vs.* LPS-stimulated cells).

Initially, we investigated the influence of TAE on the inflammatory profile of murine macrophages (*i.e.*, RAW267.4 cells) stimulated with *E. coli* lipopolysaccharide (LPS), which triggered numerous metabolic changes [10]. TAE reduced the LPS-induced production of nitric oxide (NO) and it also significantly diminished the secretion of COX-2 dependent PGE_2_ (Figure 1). Furthermore, we evaluated the effect of TAE on cytokine and chemokine (CK) production in murine macrophages. TAE concentration-dependently blunted TNF-α and IL-12(p70), while the production of anti-inflammatory IL-10 was augmented (Figure 1). Conversely, TAE had little impact on IL-1β and IL-6. Secretion of chemokines, such as CCL2/MCP-1, CCL4/MIP-1β and CCL5/RANTES, was increased by TAE (Figure 1, Table 1). We further investigated how the expression of inflammatory genes was influenced by TAE. Gene microarray analysis revealed that LPS induced robust up-regulation of hundreds of genes in RAW264.7 cells ([11] and our unpublished results). TAE diminished mRNA levels of TNF-α, IL-6, CCL4/MIP-1β, CCL5/RANTES and CXCL10/IP-10 (Figure 2). The NF-κB transcription pathway was impaired by TAE, as illustrated by reduced expression levels of NF-κB and Iκ-Ba mRNA. This suggests that TAE regulated gene expression via the NF-κB pathway (Supplementary Materials Table S1).

**Table 1:** Effects of constituents of TAE on the secretion of inflammatory metabolites by RAW264.7 macrophages. Cells were stimulated with LPS in the presence of the indicated substances and cultured for 24 h. Metabolites were determined in the culture supernatants by multiplex ELISA and Griess reaction (for nitric oxide). Representative data obtained in one of three different experimental series are shown. Mean values ± SD (of triplicate cultures) are given.

Metabolite	LPS Stimulated	TAE (500 μg/mL) +LPS	*p*	Adenosine (25 μM) +LPS	*p*	Chlorogenic Acid (25 μM) +LPS	*p*	Rutin (25 μM) +LPS	*p*
PGE_2_ [pg/mL]	7034 ± 186	5234 ± 260	0.09	6373 ± 44	0.23	12,016 ± 574	0.05	12,940 ± 1882	0.07
Nitric oxide (μM)	18.1 ± 0.2	13.1 ± 0.8	0.05	19.6 ± 0.9	0.94	12.2 ± 0.1	0.02	14.4 ± 0.2	0.04
IL-6 [ng/mL]	33.6 ± 2.4	36.9 ± 1.8	0.26	24.1 ± 2.1	0.05	27.0 ± 1.7	0.08	25.7 ± 2.0	0.07
IL-1β [pg/mL]	702 ± 120	579 ± 105	0.39	625 ± 78	0.52	567 ± 32	0.26	498 ± 22	0.14
IL-12 [pg/mL]	1083 ± 165	1045 ± 49	0.79	1150 ± 127	0.69	1058 ± 127	0.69	921 ± 35	0.31
TNF-α [ng/mL]	293 ± 33	290 ± 12	0.93	202 ± 5	0.07	241 ± 21	0.23	292 ± 10	0.98
CCL2/MCP-1 [ng/mL]	9.8 ± 1.9	14.9 ± 3.7	0.22	6.4 ± 2.9	0.30	8.8 ± 0.4	0.53	6.7 ± 1.7	0.22
CCL4/MIP-1β [pg/mL]	792 ± 50	1385 ± 63	0.01	753 ± 31	0.45	698 ± 36	0.16	793 ± 33	0.99
CCL5/RANTES [ng/mL]	34.1 ± 0.4	58.7 ± 1.5	0.002	28.6 ± 3.1	0.13	27.7 ± 0.8	0.01	28.4 ± 2.1	0.06

While TAE contained no detectable quantities of vitamin C, E and lycopene, it had significant amounts of adenosine, chlorogenic acid (CA) and rutin (Table 2), which could contribute to the altered inflammatory response. Therefore, we analyzed the impact of adenosine and the two phenolic compounds on RAW264.7 cells. We observed that adenosine significantly modulated the secretion of IL-6 and TNF-α. CA and rutin blunted NO and IL-6, whereas they had no substantial effect on the secretion of other mediators (Table 2). We also noticed differences between adenosine, CA and rutin with regard to the regulation of gene expression: adenosine and TAE had similar effects on gene regulation (Figure 2, Supplementary Materials Figure S1). Rutin and CA had a common pattern on gene expression, which only partially overlapped with that of adenosine.

**Table 2:** Constituents of tomato aqueous extracts (TAE).

Constituent	(In %) ^1^	(μM)
Adenosine	1.73	64.8
Rutin	0.592	0.96
Chlorogenic Acid	0.182	0.52
Vitamin C	<LOD ^2^	
Vitamin E	<LOD	
Lycopene	<LOD	

^1^ % of total TAE; ^2^ limits of detection.

### 2.2. TAE Altered Inflammatory Response of Peripheral Blood Leukocytes Activated by LPS

**Figure 3:**
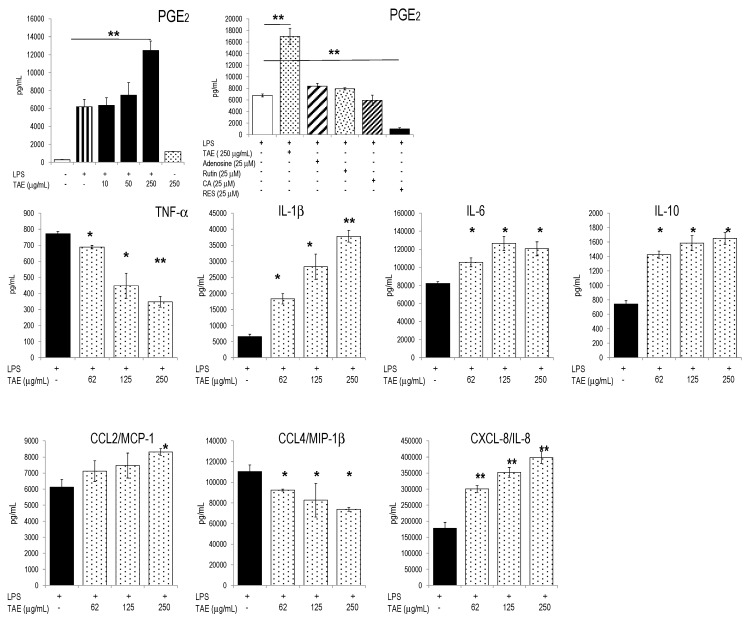
Effects of TAE on activated human PBLs. Freshly isolated PBLs were incubated with TAE, stimulated with LPS and cultured for 24 h. Representative data (±SD) of duplicates from one of three independent experiments are given. * *p* < 0.05, ** *p* < 0.01 (*vs.* LPS-stimulated cells). RES, resveratrol.

Since tomato constituents modified platelet function [6,12,13], we investigated whether TAE affected inflammatory parameters of human PBLs. To this aim, cells were stimulated with LPS in the presence of graded amounts of TAE and the inflammatory response was quantified by measuring secreted mediators and gene expression. The substances did not impair cell viability (Supplementary Materials Figure S2). In response to LPS, PBLs secreted a plethora of inflammatory mediators [10,11]. TAE increased the release of PGE_2_ by activated PBLs (Figure 3). This markedly contrasted with the strongly inhibitory effects of resveratrol, which completely suppressed PGE_2_ secretion [14]. TAE also induced significant PGE_2_ secretion in un-stimulated PBL (Supplementary Materials Figure S3). The production of IL-1β, IL-6 and IL-10 was stimulated by TAE, whereas IL-12(p70) was mitigated (Figure 3 and Supplementary Materials Table S2). With regard to CKs, TAE significantly reduced CCL4/MIP-1β secretion, but it strongly enhanced CXCL8/IL-8 production. TAE-dependent changes of cytokine and CK patterns could in part be attributed to its contents in adenosine, since adenosine modulated TNF-α and CCL4/MIP-1β in a similar way (Supplementary Materials Table S2). Neither adenosine, nor rutin or CA influenced the level of secreted PGE_2_ (Figure 3) and different ILs and CKs. When incubated with high concentrations of TAE (*i.e.*, 1 mg/mL), un-stimulated PBLs secreted significant amounts of IL-1β, IL-6, CCL2/MCP-1, CCL4/MIP-1β, CXCL8/IL-8 and PGE_2_ (Supplementary Materials Figure S3).

### 2.3. TAE Modifies Chemokine and Cytokine Gene Transcription in PBLs

**Figure 4:**
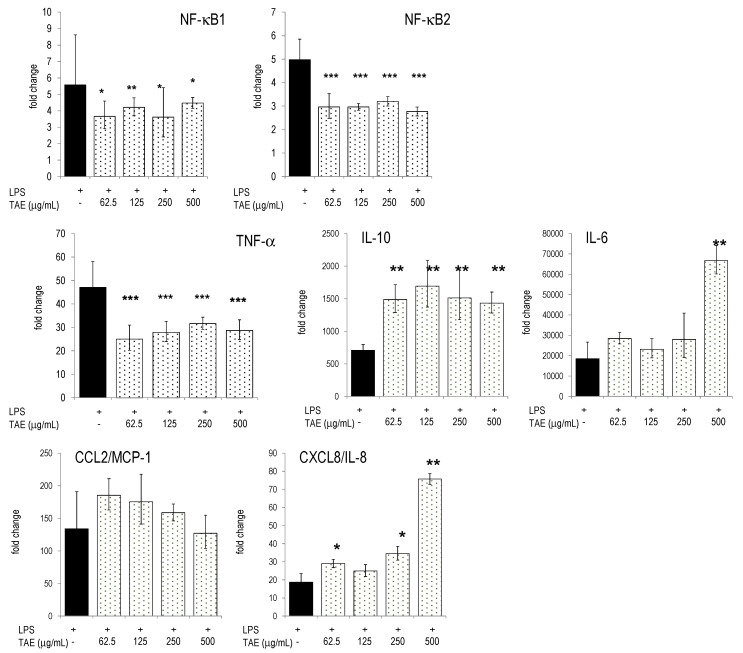
Gene expression in human PBLs and TAE-induced expression changes. Freshly isolated PBLs were incubated with TAE, stimulated with LPS and cultured for 12 h. Gene expression was quantified by RT-PCR and the data expressed as fold change compared to levels observed in unstimulated cells. Mean ± SD of duplicates are given. Representative data of one of three tested donors are shown. * *p* < 0.05, ** *p* < 0.01, *** *p* < 0.001 (*vs.* LPS-stimulated cells).

Next, we investigated expression levels of inflammatory genes in human PBLs and studied the impact of TAE. PBLs responded to LPS stimulation by the differential expression of thousands of genes ([11] and our unpublished results). Many of these genes are controlled by transcription factors (TF) of the NF-κB pathway (see e.g., [15]). Accordingly, LPS up-regulated TFs like NF-κB2 and its respective inhibitor NF-κBIα. TAE significantly down-regulated NF-κB1 and NF-κB2 (Figure 4). The levels of transcription factors, STAT1 and CREB1, were also significantly reduced by TAE (Supplementary Materials Figure S4). In addition, TAE regulated the expression levels of CK and cytokine genes which are under the control of NF-κB elements: CCL2/MCP-1 mRNA levels were lower in TAE-treated LPS-activated PBLs; conversely, CXCL8/IL-8 mRNA levels were concentration-dependently increased by TAE (Figure 4). TAE-treated PBLs had higher levels of IL-6 and IL-10 mRNA.

### 2.4. Modulation of Inflammatory Parameters Associated with Endothelial Dysfunction

**Figure 5:**
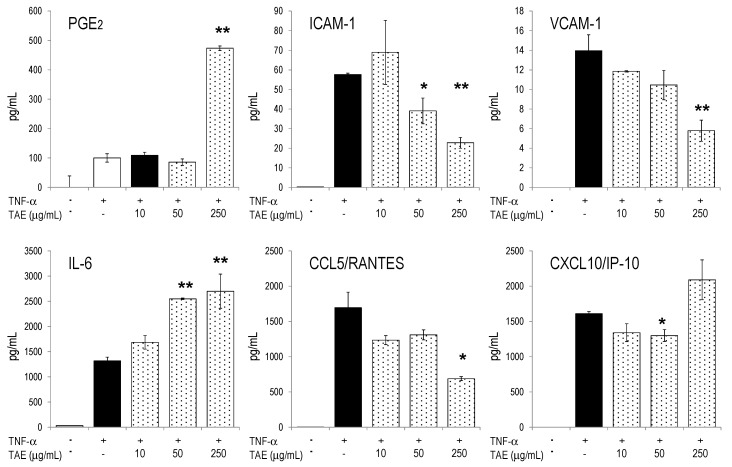
TAE influences HUVEC metabolites during endothelial dysfunction. HUVECs were incubated with TAE, activated with 10 ng/mL TNF-α and cultured for 24 h. Secreted cytokines, chemokines, ICAM-1 and VCAM-1 were quantified by Luminex technology (see Materials and Methods). Mean ± SD of duplicates are given. Unstimulated cells produced <10% of the mediators secreted by TNF-α stimulated cells. * *p* < 0.05, ** *p* < 0.01 (*vs.* TNF-α-stimulated cells).

Previous studies had shown that TAE reduced platelet aggregation and platelet adhesion to activated endothelial cells [5,6,16,17]. In order to determine the putative involvement of TAE on endothelial functions, we investigate the effect of TAE on endothelial homeostasis. To this aim, we induced endothelial dysfunction (ED) by activating HUVECs with TNF-α [18,19] and measured the impact of TAE on parameters of ED. TAE increased PGE_2_ and IL-6 production, whereas it mitigated the production of ICAM-1 and VCAM-1 (Figure 5). Similar observations were made in un-stimulated HUVECs; yet, these cells secreted only small amounts of mediators in the absence of TNF-α activation (Supplementary Materials Table S3).

**Figure 6:**
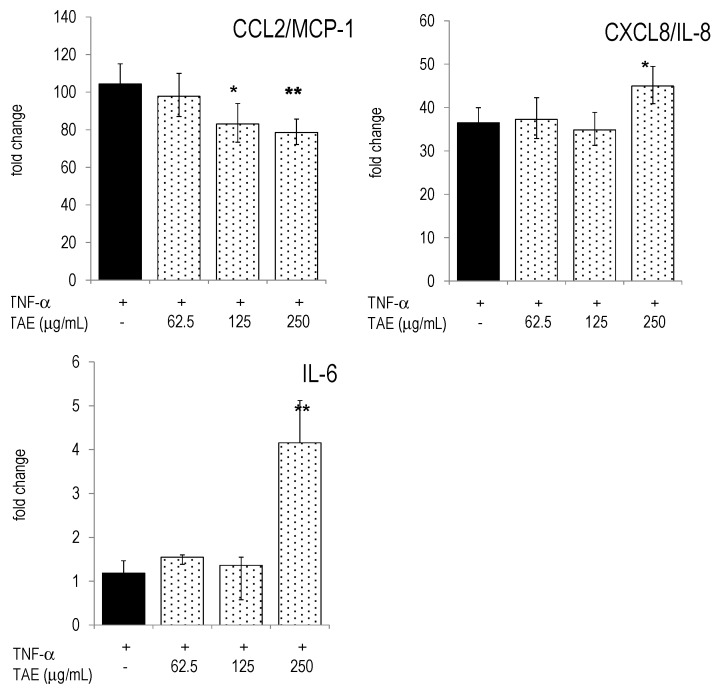

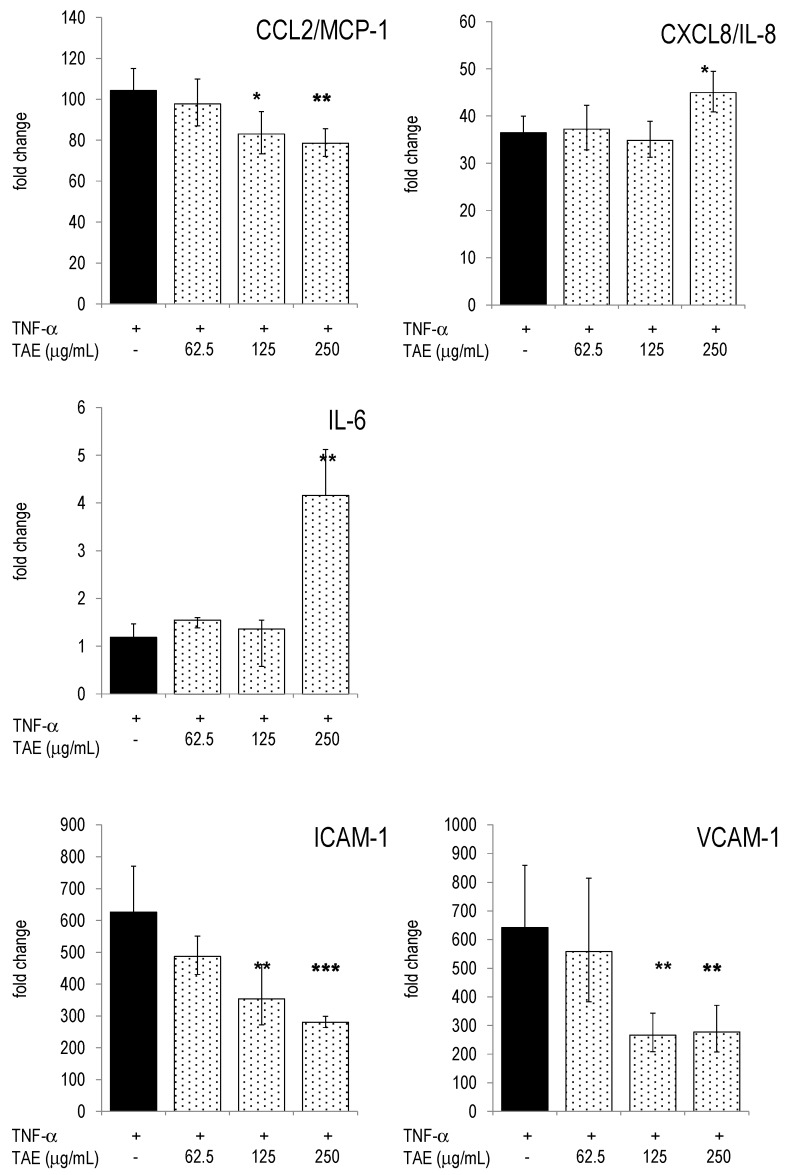
HUVECs were incubated with TAE, activated with 10 ng/mL TNF-α and cultured for 4 h. Gene expression was quantified by RT-PCR and the data expressed as fold change compared to levels observed in unstimulated cells. Mean ± standard errors of duplicates are given. * *p* < 0.05, ** *p* < 0.01, *** *p* < 0.001 (*vs.* TNF-α-stimulated cells).

TNF-α activation of HUVECs induced drastic gene expression of IL-6, ICAM-1, VCAM-1, CCL2/MCP-1 and CXCL8/IL-8 (Figure 6). TAE decreased expression levels of VCAM-1, but it increased mRNA levels for IL-6 and CXCL8/IL-8, much like the observations made with PBLs. Collectively, the effects of TAE on gene expression in TNF-α activated HUVECs matched those observed at the protein level.

## 3. Discussion

This study significantly extends our knowledge about the effects of TAE and its main constituents on the modulation of the inflammatory response in cells of the innate immune system (*i.e.*, macrophages) and the vascular-endothelial compartment (*i.e.*, PBLs and HUVECs, respectively). Previously, lipophilic constituents of tomato including carotenoids, as well as vitamins and phenolic compounds contained in tomato [8,20,21] were shown to alter biochemical and cellular features related to inflammation and oxidative status (reviewed in e.g., [9]). Consistent with the results of the present study, Bessler *et al.* reported that in the presence of lycopene *in vitro* stimulated PBLs increased the secretion of IL-1β and TNF-α, while IL-10 and IFN-γ was impaired [8]. More specifically, CA and rutin impeded the production of pro-inflammatory cytokines in macrophages via the NF-κB signalling pathway [22,23,24,25]. Presumably, they marginally contributed to the biological profile of TAE described in this study, because their concentration in TAE was <1 μM. On the other hand, TAE contained significant amounts of adenosine, which reduced the production of TNF-α, IL-6 and different CKs, and it down-regulated NF-κB1 gene expression. The results of this study suggest that adenosine was the major, but not unique bio-active compound of TAE. As shown by others [26,27,28], adenosine modulated the inflammatory response in immune and endothelial cells and it regulated interleukin expression both transcriptionally and post-transcriptionally. However, adenosine differed from TAE in some inflammatory parameters: it did not alter PGE_2_ production in PBLs, while TAE enhanced it (Supplementary Materials Table S2). Other notable exceptions were observed in the expression and secretion of IL-1β, CCL2/MCP-1, CXCL8/IL-8, where adenosine opposed the effects to TAE. Adenosine impaired CCL2/MCP-1 and CCL4/MIP-1β and thus trafficking of monocytes and neutrophils and influenced the M1/M2 polarization and function of macrophages [29,30].

Inflammatory processes are tightly regulated by CKs and cytokines. CKs orchestrate the migration and trafficking of cells into different compartments of the immune system. Monocytes, which play key roles in the innate immune response, are recruited by CCL2/MCP-1, CCL3/MIP-1α and CCL4/MIP-1β. Activated T cells are involved in the adaptive immune response and are chemo-attracted by CCL5/RANTES and CXCL10/IP-10. Neutrophils are recruited by CXCL8/IL-8 [31], which is produced during sterile inflammation [32]. In the blood compartment (*i.e.*, PBLs), TAE dampened the production of CCL4/MIP-1β, but enhanced CXCL8/IL-8. It should be emphasized that a given metabolite can be regulated in a context-dependent way. This has been compellingly shown for IL-6, which controls survival and differentiation of B and T lymphocytes, whereas it favors inflammatory processes in, and thus impairs survival of malignant cells [33]. PBLs comprise distinct cell populations including lymphocytes, monocytes/macrophages and neutrophils, which are key cellular actors in the inflammatory response and each of which produces different levels of IL-6 upon activation. It remains to be investigated whether these cell populations have idiosyncratic responses to TAE.

Upon TNF-α stimulation HUVECs expressed a pattern of cytokines and chemokines, which had many common features with that of LPS-activated PBLs. In endothelial cells TAE modulated cytokine expression in a similar way as in PBLs. Remarkably, TAE reversed the expression of ICAM-1 and VCAM-1 towards pre-ED homeostasis (Figure 5 and [6]). Thus, reduced endothelial diapedesis might be one biological consequence of the presence of the substances in the blood. Adenosine had similar effects on HUVECs and might therefore largely account for its biological efficacy in the endothelial layer [34,35].

**Figure 7:**
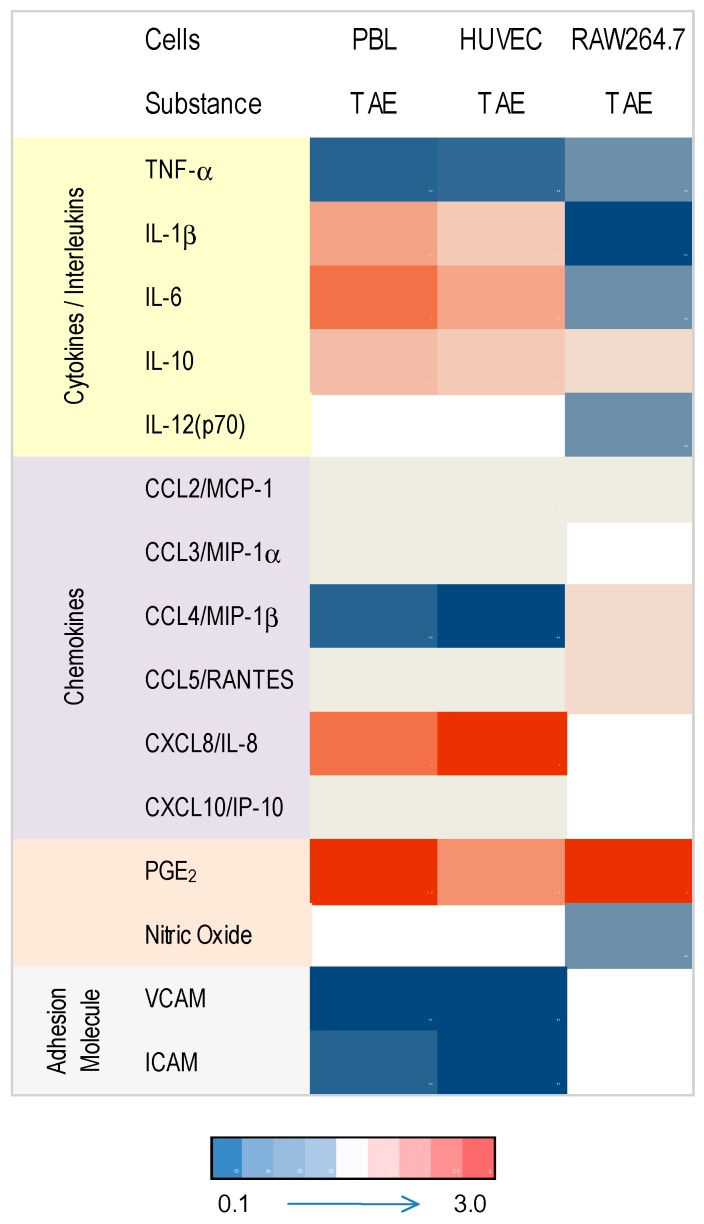
Heat map depicting regulation of inflammatory parameters by TAE across immune and endothelial cells. Values were calculated relative to the metabolite expression or secretion observed in stimulated cells. Intensity of blue and red color grades indicate reduced and increased expression of metabolites, respectively. Empty fields: no data available or values below limit of detection).

**Figure 8:**
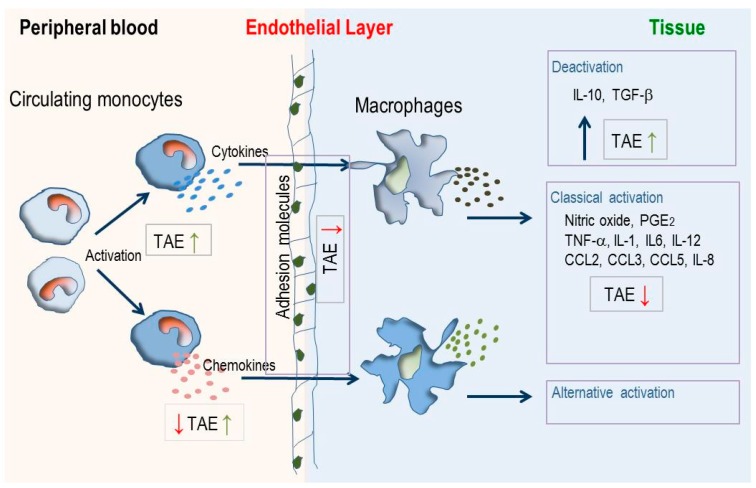
Modulation of inflammatory processes in blood and tissue by TAE. Circulating monocytes respond to activation signals (like pathogen associated molecular patterns, Toll-like receptor ligands, lipopolysaccharides) by increased production of cytokines and chemokines, migrate through endothelial layers and differentiate into tissue-resident macrophages. These can be stimulated by the classical or alternative activation pathway. TAE enhances (green arrows) or diminishes (red arrows) cytokines, chemokines and adhesion molecules which orchestrate cell trafficking, activation and differentiation in different compartments of the immune system.

Macrophages, which develop from monocytes, are pivotal during initiation and resolution of inflammation and they differentiate into tissue-specific subsets [16,36]. TAE regulated cytokine and interleukin production by macrophages, suggesting that it dampened the classical activation by LPS (IL-1β, IL-6, IL-12) and favored alternative activation via IL-10 (Figure 7 and Figure 8). With regard to CKs, the tested substance had a lesser impact on macrophages as compared to PBLs: TAE increased CCL4/MIP-1β and CCL5/RANTES and therefore recruitment of monocyte and activated T lymphocytes. Although the data rely on experiments obtained with a murine macrophage cell line, we infer that the observations are not species-restricted. Indeed, similar observations were made with macrophages that were differentiated from human peripheral blood mononuclear cells (our unpublished results).

The molecular regulation of inflammatory processes is dependent on numerous transcription factors (TF). Notably, LPS-activation of monocytes and macrophages induced the NF-κB dependent transcription of chemokines, such as CXCL8, CXCL10, and CCL2 (reviewed in [37]), whereas IL-4 and IL-10 inhibited IFN-γ dependent CXCL10 and CCL5 transcription [38,39]. Here, we show that the transcription levels of NF-κB elements, as well as STAT1 and CREB1, were influenced by TAE. Further investigations should focus on the putative specific effects of TAE on TF in different cells and tissues. Additionally, it needs to be shown that TAE impairs the NF-κB pathway also at the post-transcriptional level.

The clinical relevance of the present *in vitro* data need to be addressed in a nutritional intervention trial. As shown in a seminal study by Bakker *et al.* [4], supplementation of overweight subjects for five weeks with dietary products including resveratrol, green tea extract, tomato extract, vitamin C, α-tocopherol and PUFAs modified some plasma metabolites. Subtle changes in expression levels of IL-12, ICAM-1 and VCAM-1 were also observed and were consistent with the data of this study. In contrast, Bakker *et al.* did not notice *in vivo* changes of CKs similar to those described here for CCL2/MCP-1, CCL3/MIP-1α, CCL5/RANTES, CXCL8/IL-8 or PGE_2_. Presumably, at conditions of prolonged supplementation, systemic changes induced by nutrients did no longer induce quantifiable metabolic changes. Hence the impact of substances remained undetected. This contrasts with the data described in this work, where the experimental set-up represents acute inflammation and short term supplementation and it precludes metabolic transformation and transport of nutrients. We hypothesize that measurable effects of nutrients on plasma metabolites or leukocyte gene expression will only be detected *in vivo* at conditions of immediately perturbed homeostasis subsequent to food intake [40]. Interestingly, TAE had measurable *in vivo* effects on platelet aggregation and adhesion in individuals who were supplemented with a single dose of the tomato extracts [6,13]. This prompts us to anticipate that TAE will also have effects in clinical studies where inflammatory parameters will be measured.

A synopsis of the different features is shown in Figure 7 and Figure 8. TAE had idiosyncratic effects on PBLs, HUVECs and macrophages. TAE modified ILs and TNF-α along a cell-specific boundary. The nutrient favored production of IL-1β, IL-6 and IL-12 in activated PBL and HUVEC, but inhibited them in RAW264.7 cells. Increased IL-1β, IL-6 or IL-12 is commonly associated with a pro-inflammatory status. T_h_ lymphocyte differentiation, however, depends on these ILs in the adaptive immune response (AIR) [41,42,43]. Consequently, immune cells transiently exposed to TAE would produce low levels of cytokines and, thus, be primed for AIR. Moreover, IL-6 regulates T_h_ lymphocyte differentiation since it promotes T_h_2 and inhibits T_h_1 differentiation by two independent molecular mechanisms [43]. It is also required for alternative activation of macrophages [44,45] and antibody class switching. Presumably, the constituents of TAE have a short plasma half-life comparable to other nutrients [46] and, thus, prime immune cells only briefly in the blood. Therefore, the TAE-induced increase of alertness is high in the periphery, but low at the boundaries of the vascular compartment and reverted in macrophages, which participate in chronic inflammation. TAE might therefore contribute to improve the systemic response to “danger” [3,47,48] and concomitantly reduce the low-grade inflammatory status related to chronic diseases. This set of data further strengthens the concept that multi-parametric metabolic analyses leads to a context-dependent profiling of TAE. It further indicates that the effect of TAE ought to be characterized and interpreted through contextualization and analysis of variables in different biological conditions.

## 4. Materials and Methods

### 4.1. Reagents

TAE was prepared from a proprietary blend of Lycopersicon esculentum containing five tomato cultivars (Heinz, Jet, Falcorosso, Early Fire, Premium 2000) as described by [6]. Briefly, ripe tomato fruits of *Lycopersicon esculentum* were homogenized, cleared by centrifugation and ultra-filtered (MW cutoff 1000 Da). Biologically inactive material, which mainly consisted of soluble sugars, was removed by solid-phase extraction; non-sugar components (4% of aqueous extract dry matter) were eluted in ethanol. The dried eluate was dissolved in DMSO. Its main constituents are indicated in Table 1. Adenosine, rutin, chlorogenic acid (CA), *E. coli* lipopolysaccharide (LPS, serotype 055:B5) and fetal bovine serum (FBS) were from Sigma/Aldrich (Saint-Louis, MO, USA). RPMI 1640, DMEM, 2-mercaptoethanol and non-essential amino acids (NEAA) were from Invitrogen (Carlsbad, CA, USA). TNF-α was from PeproTech EC (London, UK).

### 4.2. Cell Culture

RAW264.7 cells and human PBLs have been cultured and treated with inflammatory stimuli as described [12,13,14]. Briefly, PBLs were obtained from healthy donors and cultured (at 8 × 10^6^ viable cells/mL) in phenol-red free RPMI 1640 (containing 0.25% FBS, 0.1 mM NEAA, 50 U/mL penicillin, 50 μg/mL streptomycin, 50 μM 2-mercaptoethanol). Cells were stimulated with LPS (100 ng/mL) in the presence of graded amounts of test substances. Cells were lysed in RLT buffer (Qiagen, Hilden, Germany) after 12 h of culture and total RNA was extracted. For the analysis of secreted mediators and proteins, cells were cultured for 24 h; supernatants were then collected and stored at −80 °C until use.

HUVECs were from Lonza, (Walkersville, MD, USA) and cultured in EGM (Endothelial Growth Medium, Lonza). They were used for experiments between passages 3 to 7. Cells ( at 2 × 10^5^ per well) were seeded into BioCoat™ Collagen I 6-well plates (Becton Dickinson, San Jose, CA, USA), activated with 10 ng/mL TNF-α and cultured for 4–24 h. All treatments were done in duplicate and all experimental series were done at least twice.

### 4.3. RNA Isolation, cDNA Synthesis and RT-PCR

The isolation of total RNA, synthesis of cDNA and quantitative RT-PCR has been performed as detailed before [14].

### 4.4. Multiparametricanalysis of Cytokines, Chemokines and Interleukins

The kits for the quantification of chemokines, cytokines and interleukins were purchased from BIO-RAD Laboratories (Hercules, CA, USA) and used in the LiquiChip Workstation IS 200 (Qiagen, Hilden, Germany). Data evaluation was made with the LiquiChip Analyser software (Qiagen) [49]. Nitric oxide (NO) and prostaglandin E_2_ (PGE_2_) were measured as described previously [14,49].

### 4.5. Statistical Analysis

Data were evaluated by statistical tools described previously [49]. A *p* value < 0.05 (calculated by using Student’s t test or one-way ANOVA) was considered to reflect statistically significant differences.

## 5. Conclusions

TAE interacts with immune cells and endothelial cells. It distinctly modulates the production of inflammatory modulators in a context-specific way and *in extenso* presumably in different body compartments. Consequently, TAE may beneficially enhance and attenuate inflammatory processes during acute and chronic inflammation, respectively.

## Supplementary Materials

Supplementary materials can be accessed at: http://www.mdpi.com/1420-3049/21/2/168/s1.

## Abbreviations

The following abbreviations are used in this manuscript:

CA: chlorogenic acid
CK: chemokine
ED: endothelial dysfunction
HUVECs: human umbilical vein endothelial cells
IL: interleukin
NO: nitric oxide
PGE_2_: prostaglandin E_2_
PBLs: peripheral blood leukocytes
TAE: tomato aqueous extract
TF: transcription factor

## References

[1] Hotamisligil G.S., Erbay E. (2008). Nutrient sensing and inflammation in metabolic diseases. Nat. Rev. Immunol..

[2] Serhan C.N., Savill J. (2005). Resolution of inflammation: The beginning programs the end. Nat. Immunol..

[3] Chawla A., Nguyen K.D., Goh Y.P. (2011). Macrophage-mediated inflammation in metabolic disease. Nat. Rev. Immunol..

[4] Bakker G.C., van Erk M.J., Pellis L., Wopereis S., Rubingh C.M., Cnubben N.H., Kooistra T., van Ommen B., Hendriks H.F. (2010). An antiinflammatory dietary mix modulates inflammation and oxidative and metabolic stress in overweight men: A nutrigenomics approach. Am. J. Clin. Nutr..

[5] Fuentes E.J., Astudillo L.A., Gutierrez M.I., Contreras S.O., Bustamante L.O., Rubio P.I., Moore-Carrasco R., Alarcon M.A., Fuentes J.A., Gonzalez D.E. (2012). Fractions of aqueous and methanolic extracts from tomato (*Solanum lycopersicum* L.) present platelet antiaggregant activity. Blood Coagul. Fibrinolysis.

[6] O’Kennedy N., Crosbie L., van Lieshout M., Broom J.I., Broom J.I., Webb D.J., Duttaroy A.K. (2006). Effects of antiplatelet components of tomato extract on platelet function *in vitro* and *ex vivo*: A time-course cannulation study in healthy humans. Am. J. Clin. Nutr..

[7] Rodriguez-Azua R., Treuer A., Moore-Carrasco R., Cortacans D., Gutierrez M., Astudillo L., Fuentes E., Palomo I. (2014). Effect of tomato industrial processing (different hybrids, paste, and pomace) on inhibition of platelet function *in vitro*, *ex vivo*, and *in vivo*. J. Med. Food.

[8] Bessler H., Salman H., Bergman M., Alcalay Y., Djaldetti M. (2008). *In vitro* effect of lycopene on cytokine production by human peripheral blood mononuclear cells. Immunol. Investig..

[9] Raiola A., Rigano M.M., Calafiore R., Frusciante L., Barone A. (2014). Enhancing the health-promoting effects of tomato fruit for biofortified food. Mediat. Inflamm..

[10] Seok J., Warren H.S., Cuenca A.G., Mindrinos M.N., Baker H.V., Xu W., Richards D.R., McDonald-Smith G.P., Gao H., Hennessy L. (2013). Genomic responses in mouse models poorly mimic human inflammatory diseases. Proc. Natl. Acad. Sci. USA.

[11] Takao K., Miyakawa T. (2015). Genomic responses in mouse models greatly mimic human inflammatory diseases. Proc. Natl. Acad. Sci. USA.

[12] Armoza A., Haim Y., Bashiri A., Wolak T., Paran E. (2013). Tomato extract and the carotenoids lycopene and lutein improve endothelial function and attenuate inflammatory NF-kappaB signaling in endothelial cells. J. Hypertens..

[13] O’Kennedy N., Crosbie L., Whelan S., Luther V., Horgan G., Broom J.I., Webb D.J., Duttaroy A.K. (2006). Effects of tomato extract on platelet function: A double-blinded crossover study in healthy humans. Am. J. Clin. Nutr..

[14] Richard N., Porath D., Radspieler A., Schwager J. (2005). Effects of resveratrol, piceatannol, tri-acetoxystilbene, and genistein on the inflammatory response of human peripheral blood leukocytes. Mol. Nutr. Food Res..

[15] Gasparini C., Feldmann M. (2012). NF-kappaB as a target for modulating inflammatory responses. Curr. Pharm. Des..

[16] Biswas S.K., Mantovani A. (2010). Macrophage plasticity and interaction with lymphocyte subsets: Cancer as a paradigm. Nat. Immunol..

[17] Ostertag L.M., O’Kennedy N., Kroon P.A., Duthie G.G., de Roos B. (2010). Impact of dietary polyphenols on human platelet function—A critical review of controlled dietary intervention studies. Mol. Nutr. Food Res..

[18] Furie M.B., McHugh D.D. (1989). Migration of neutrophils across endothelial monolayers is stimulated by treatment of the monolayers with interleukin-1 or tumor necrosis factor-alpha. J. Immunol..

[19] May L.T., Torcia G., Cozzolino F., Ray A., Tatter S.B., Santhanam U., Sehgal P.B., Stern D. (1989). Interleukin-6 gene expression in human endothelial cells: RNA start sites, multiple IL-6 proteins and inhibition of proliferation. Biochem. Biophys. Res. Commun..

[20] Kim Y.I., Mohri S., Hirai S., Lin S., Goto T., Ohyane C., Sakamoto T., Takahashi H., Shibata D., Takahashi N. (2015). Tomato extract suppresses the production of proinflammatory mediators induced by interaction between adipocytes and macrophages. Biosci. Biotechnol. Biochem..

[21] Simone R.E., Russo M., Catalano A., Monego G., Froehlich K., Boehm V., Palozza P. (2011). Lycopene inhibits NF-κB-mediated IL-8 expression and changes redox and PPARgamma signalling in cigarette smoke-stimulated macrophages. PLoS ONE.

[22] Gao M., Ma Y., Liu D. (2013). Rutin suppresses palmitic acids-triggered inflammation in macrophages and blocks high fat diet-induced obesity and fatty liver in mice. Pharm. Res..

[23] Shan J., Fu J., Zhao Z., Kong X., Huang H., Luo L., Yin Z. (2009). Chlorogenic acid inhibits lipopolysaccharide-induced cyclooxygenase-2 expression in RAW264.7 cells through suppressing NF-kappaB and JNK/AP-1 activation. Int. Immunopharmacol..

[24] Shi H., Dong L., Jiang J., Zhao J., Jiang J., Wang Y., Lu X., Guo X. (2013). Chlorogenic acid reduces liver inflammation and fibrosis through inhibition of toll-like receptor 4 signaling pathway. Toxicology.

[25] Wu C.H., Wu C.F., Huang H.W., Jao Y.C., Yen G.C. (2009). Naturally occurring flavonoids attenuate high glucose-induced expression of proinflammatory cytokines in human monocytic THP-1 cells. Mol. Nutr. Food Res..

[26] Hasko G., Cronstein B. (2013). Regulation of inflammation by adenosine. Front. Immunol..

[27] Hasko G., Kuhel D.G., Chen J.F., Schwarzschild M.A., Deitch E.A., Mabley J.G., Marton A., Szabo C. (2000). Adenosine inhibits IL-12 and TNF-α production via adenosine A2a receptor-dependent and independent mechanisms. FASEB J..

[28] Nemeth Z.H., Lutz C.S., Csoka B., Deitch E.A., Leibovich S.J., Gause W.C., Tone M., Pacher P., Vizi E.S., Hasko G. (2005). Adenosine augments IL-10 production by macrophages through an A2B receptor-mediated posttranscriptional mechanism. J. Immunol..

[29] Barczyk K., Ehrchen J., Tenbrock K., Ahlmann M., Kneidl J., Viemann D., Roth J. (2010). Glucocorticoids promote survival of anti-inflammatory macrophages via stimulation of adenosine receptor A3. Blood.

[30] Hasko G., Szabo C., Nemeth Z.H., Kvetan V., Pastores S.M., Vizi E.S. (1996). Adenosine receptor agonists differentially regulate IL-10, TNF-alpha, and nitric oxide production in RAW264.7 macrophages and in endotoxemic mice. J. Immunol..

[31] Baggiolini M. (1998). Chemokines and leukocyte traffic. Nature.

[32] Phillipson M., Kubes P. (2011). The neutrophil in vascular inflammation. Nat. Med..

[33] Hunter C.A., Jones S.A. (2015). IL-6 as a keystone cytokine in health and disease. Nat. Immunol..

[34] Hassanian S.M., Dinarvand P., Rezaie A.R. (2014). Adenosine regulates the proinflammatory signaling function of thrombin in endothelial cells. J. Cell. Physiol..

[35] Kaneider N., Mosheimer B., Reinisch N., Patsch J.R., Wiedermann C.J. (2004). Inhibition of thrombin-induced signaling by resveratrol and quercetin: Effects on adenosine nucleotide metabolism in endothelial cells and platelet-neutrophil interactions. Thromb. Res..

[36] Gordon S., Taylor P.R. (2005). Monocyte and macrophage heterogeneity. Nat. Rev. Immunol..

[37] Richmond A. (2002). Nf-kappa B, chemokine gene transcription and tumour growth. Nat. Rev. Immunol..

[38] Ohmori Y., Hamilton T.A. (2001). Requirement for STAT1 in LPS-induced gene expression in macrophages. J. Leukoc. Biol..

[39] Ito S., Ansari P., Sakatsume M., Dickensheets H., Vazquez N., Donnelly R.P., Larner A.C., Finbloom D.S. (1999). Interleukin-10 inhibits expression of both interferon alpha- and interferon gamma- induced genes by suppressing tyrosine phosphorylation of STAT1. Blood.

[40] Baty F., Facompre M., Wiegand J., Schwager J., Brutsche M.H. (2006). Analysis with respect to instrumental variables for the exploration of microarray data structures. BMC Bioinform..

[41] Diehl S., Rincon M. (2002). The two faces of IL-6 on Th1/Th2 differentiation. Mol. Immunol..

[42] Heusinkveld M., de Vos van Steenwijk P.J., Goedemans R., Ramwadhdoebe T.H., Gorter A., Welters M.J., van Hall T., van der Burg S.H. (2011). M2 macrophages induced by prostaglandin E2 and IL-6 from cervical carcinoma are switched to activated M1 macrophages by CD4+ Th1 cells. J. Immunol..

[43] Mayer A., Debuisson D., Denanglaire S., Eddahri F., Fievez L., Hercor M., Triffaux E., Moser M., Bureau F., Leo O. (2014). Antigen presenting cell-derived IL-6 restricts Th2-cell differentiation. Eur. J. Immunol..

[44] Mantovani A., Sozzani S., Locati M., Allavena P., Sica A. (2002). Macrophage polarization: Tumor-associated macrophages as a paradigm for polarized M2 mononuclear phagocytes. Trends Immunol..

[45] Mauer J., Chaurasia B., Goldau J., Vogt M.C., Ruud J., Nguyen K.D., Theurich S., Hausen A.C., Schmitz J., Bronneke H.S. (2014). Signaling by IL-6 promotes alternative activation of macrophages to limit endotoxemia and obesity-associated resistance to insulin. Nat. Immunol..

[46] Walle T., Hsieh F., DeLegge M.H., Oatis J.E., Walle U.K. (2004). High absorption but very low bioavailability of oral resveratrol in humans. Drug Metab. Dispos..

[47] Gallucci S., Matzinger P. (2001). Danger signals: SOS to the immune system. Curr. Opin. Immunol..

[48] Skoberne M., Beignon A.S., Bhardwaj N. (2004). Danger signals: A time and space continuum. Trends Mol. Med..

[49] Schwager J., Hoeller U., Wolfram S., Richard N. (2011). Rose hip and its constituent galactolipids confer cartilage protection by modulating cytokine, and chemokine expression. BMC Complement. Altern. Med..

