# Supplementary material for: Tomato Aqueous Extract Modulates the Inflammatory Profile of Immune Cells and Endothelial Cells
Source: Molecules. 2016 Jan 29;21(2):168. doi: 10.3390/molecules21020168 (PMC6273921; doi:10.3390/molecules21020168)
Supplement: Supplementary file 1 [file molecules-21-00168-s001.pdf]

Supplementary Materials: Tomato Aqueous Extract Modulates the Inflammatory Profile of Immune Cells and Endothelial Cells

Joseph Schwager, Nathalie Richard, Bernd Mussler and Daniel Raederstorff

Table S1. Effect of TAE on expression of genes in RAW264.7 cells. RAW264.7 cells were stimulated with LPS in the presence of TAE and cultured for 4 h. Gene expression was quantified by RT-PCR. Representative data obtained in one of three different experimental series are shown. Mean values ± SD (of duplicate) are given.

Gene	Fold Change LPS Stimulated	Fold Change TAE (500 µg/mL) +LPS-Stimulated	*p* Value
IL-6	15,259 ± 2673	10,712 ± 3081	0.06
IL-10	35.3 ± 4.8	46.3 ± 5.6	0.06
TNF-α	35.2 ± 3.4	30.9 ± 1.4	0.06
CXCL10/IP-10	279 ± 32	169 ± 32	0.003
NF-κB49	6.3 ± 0.7	4.4 ± 0.5	0.002
NF-κB1	9.8 ± 1.3	7.5 ± 1.0	0.03
Iκ-Ba	10.0 ± 0.7	8.3 ± 1.4	0.07

Table S2. Effects of TAE and its main constituents on the secretion of inflammatory metabolites by PBLs. Freshly isolated PBLs were stimulated with LPS in the presence of the indicated substances and cultured for 24 h. Metabolites were determined in the culture supernatants by multiplex ELISA. Representative data obtained from PBLs of one of three different donors are shown. Mean values ± SD (of duplicate cultures) are given.

Metabolite	LPS Stimulated	TAE (500 μg/mL) +LPS	*p*	Adenosine (25 μM) +LPS	*p*	CA (25 μM) +LPS	*p*	Rutin (25 μM) +LPS	*p*
PGE_2 [pg/mL]	6787 ± 252	20622 ± 2493	0.01	8385 ± 413	0.79	5923 ± 897	0.21	7914 ± 187	0.32
IL-6 [ng/mL]	116.0 ± 11.3	134.0 ± 7.1	0.20	100.1 ± 11.1	0.29	92.5 ± 6.9	0.12	110.5 ± 13.4	0.70
IL-1β [ng/mL]	17.5 ± 3.0	41.4 ± 4.2	0.01	13.5 ± 1.9	0.66	8.9 ± 0.1	0.11	11.1 ± 1.8	0.29
IL-12 [pg/mL]	27 ± 0	23 ± 2	0.10	29 ± 5	0.62	27 ± 7	0.98	30.0 ± 1.0	0.09
TNF-α [pg/mL]	3870 ± 993	751 ± 52	0.08	609 ± 42	0.11	1045 ± 64	0.07	1485 ± 163	0.09
CCL2/MCP-1 [pg/mL]	913 ± 166	648 ± 407	0.48	472 ± 103	0.09	810 ± 298	0.71	670 ± 187	0.30
CCL4/MIP-1β [ng/mL]	116.5 ± 16.2	32.6 ± 0.8	0.06	72.2 ± 0.9	0.06	81.3 ± 6.7	0.11	99.8 ± 3.1	0.29
CCL5/RANTES [pg/mL]	771 ± 51	1050 ± 42	0.02	825 ± 27	0.21	928 ± 131	0.22	872 ± 120	0.32

Table S3. Effects of TAE and adenosine on the secretion of inflammatory metabolites by unstimulated HUVEC. Human umbilical vein endothelial cells were cultured in the presence of graded amounts of TAE or adenosine for 24 h. The amount of metabolites in the culture supernatant was determined by EIA and multiplex ELISA. Mean values ± SD (of duplicate cultures) are given.

Metabolite	Unstimulated	TAE (250 µmg/mL)	Adenosine (50 µM)
PGE_2	55 ± 29	71 ± 28	75 ± 40
IL-6	30 ± 1	83 ± 9	33 ± 2
CCL2/MCP-1	291 ± 57	637 ± 42	261 ± 5
CCL5/RANTES	3 ± 1	4 ± 0	4 ± 1
CXCL8/IL-8	1345 ± 148	2110 ± 240	1450 ± 0
CXCL10/IP-10	1 ± 0	6 ± 3	2 ± 0
VCAM	0 ± 0	0 ± 0	0 ± 0
ICAM	0 ± 0	0 ± 0	0 ± 0

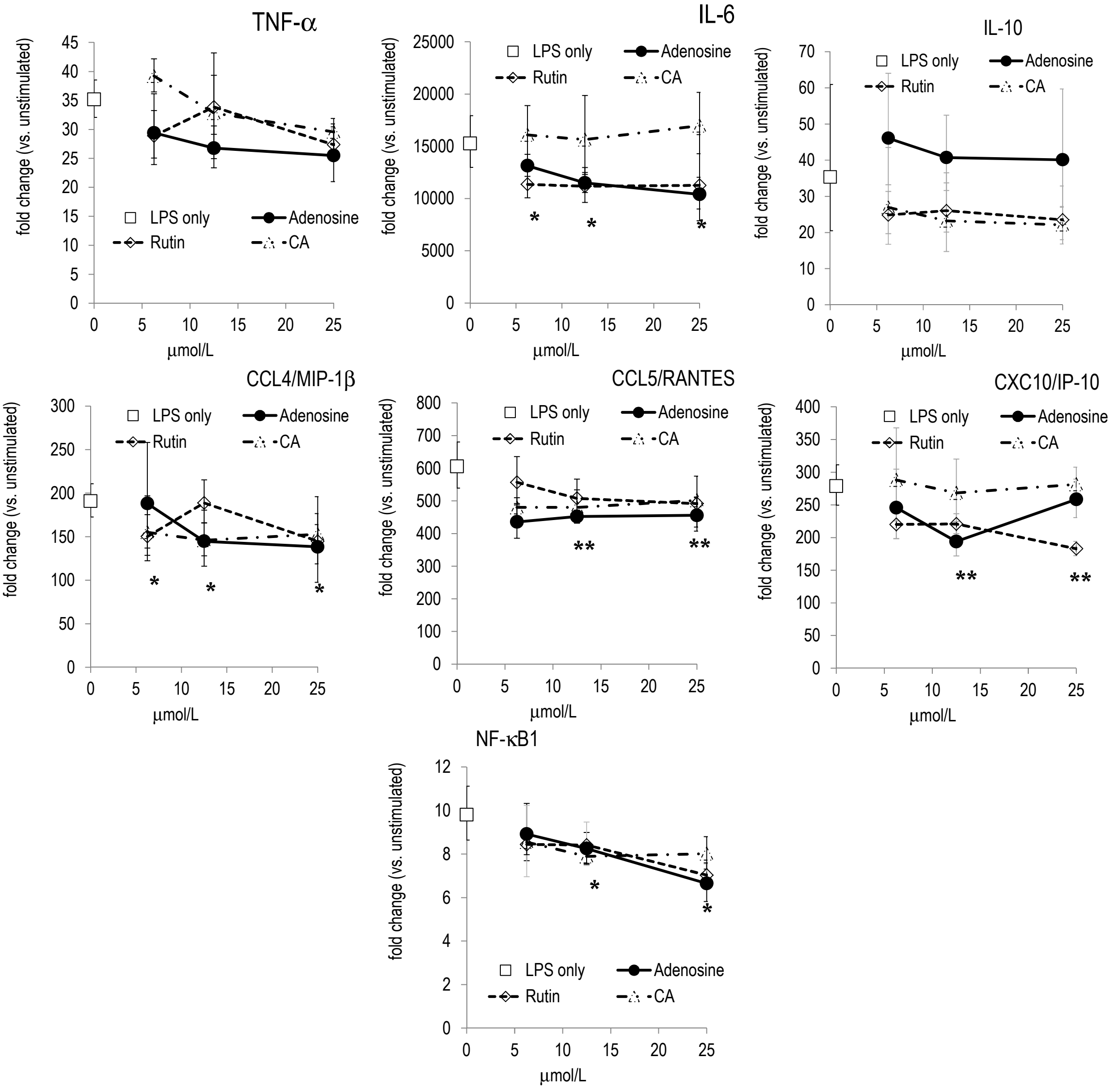

Figure S1. Impact of TAE constituents adenosine, rutin and chlorogenic acid (CA) on cytokine and chemokine expression. RAW264.7 cells were stimulated with LPS in the presence of a graded amounts of adenosine, rutin and CA for 4 h. Gene expression was quantified by RT-PCR. Representative data obtained from one of two different experimental series are shown. Mean values ± SD (of duplicate) are given. * $p < 0.05$, ** $p < 0.01$ (*vs.* LPS-stimulated cells).

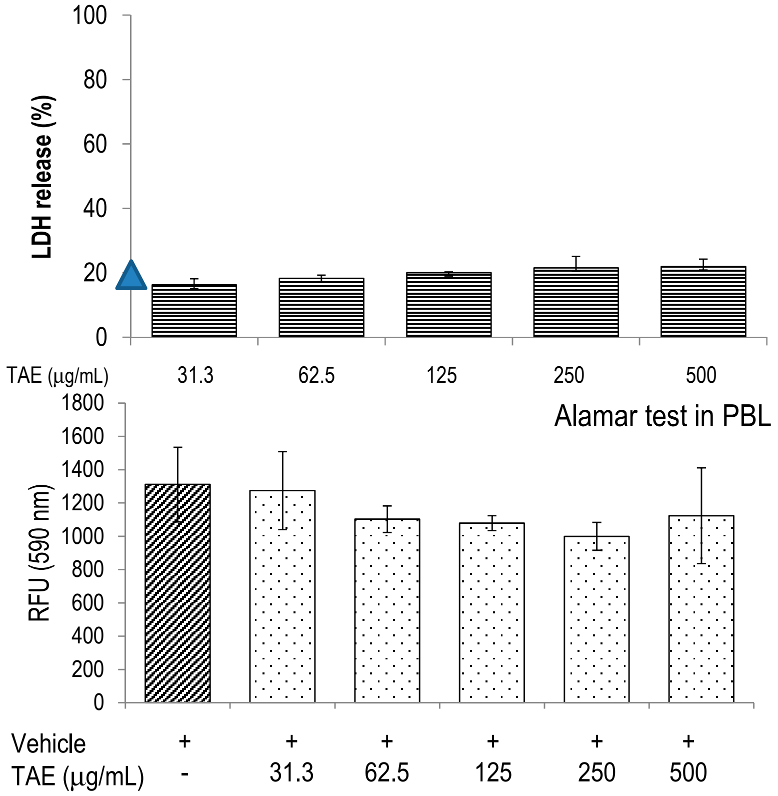

Figure S2. Viability of PBLs is not affected by TAE. Freshly isolated PBLs were incubated with the indicated amounts tomato aqueous extract (TAE) for 24 h. LDH released into the culture medium was measured with the commercially available viability assay systems (Promega) (**upper panel**) and is expressed as % of total cellular LDH. The symbol on the y-axis indicates the value obtained with cells that were not exposed to TAE. Alternatively, the Alamar Blue® cell viability test was applied (**lower panel**) following the instructions of the manufacturer (ThermoFischer Scientific).

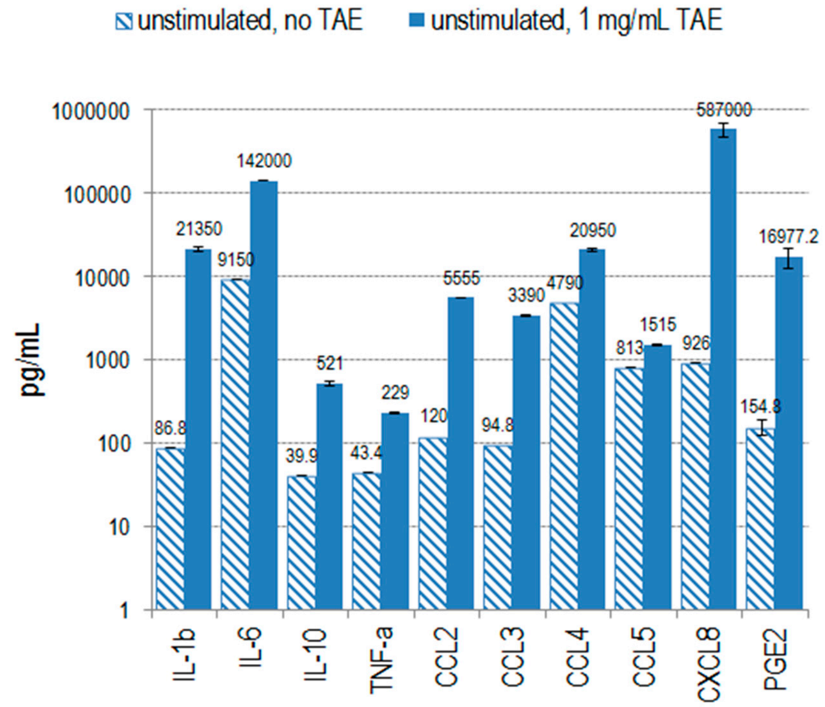

Figure S3. Production of PGE_2, cytokines and chemokines by unstimulated PBLs. Freshly isolated PBLs were cultured for 24 h without or with TAE (1 mg/mL). Secreted metabolites were determined by multiplex analysis. Mean-values (±SD) of duplicate cultures are given. Similar observations were made with PBLs obtained from five different donors.

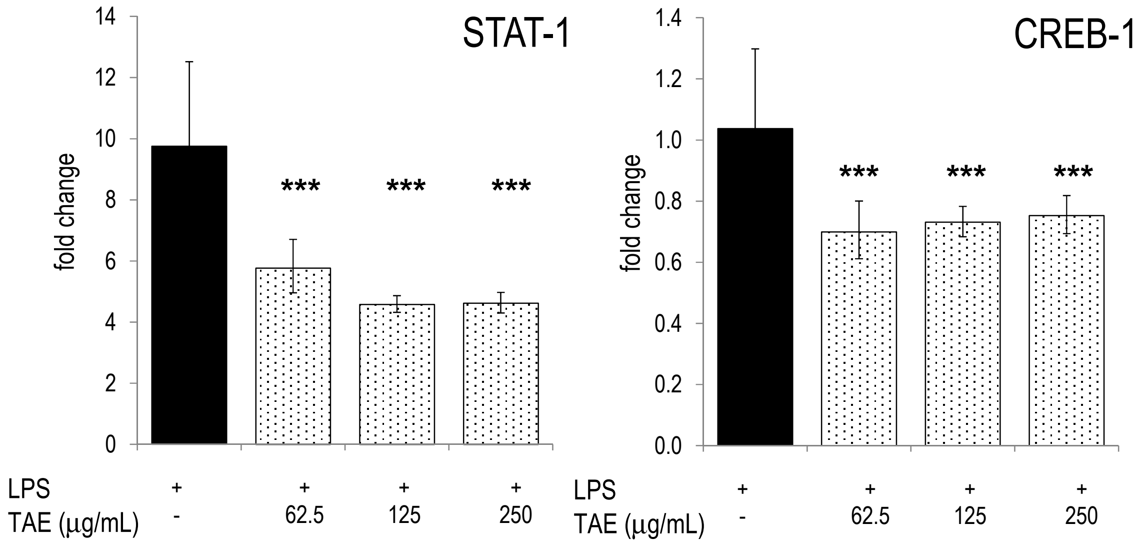

Figure S4. Expression of STAT1 and CREB1 in PBL. Freshly isolated PBLs were incubated with the indicated amounts of tomato aqueous extract (TAE) for 4 h. Gene expression was quantified by RT-PCR. Mean values ± SD (of duplicate) are given. Representative data obtained from one of three donors are shown. *** $p < 0.001$ (*vs.* LPS-stimulated cells).
